# Incremental Value of Radiomics in 5-Year Overall Survival Prediction for Stage II–III Rectal Cancer

**DOI:** 10.3389/fonc.2022.779030

**Published:** 2022-06-30

**Authors:** Ke Nie, Peng Hu, Jianjun Zheng, Yang Zhang, Pengfei Yang, Salma K. Jabbour, Ning Yue, Xue Dong, Shufeng Xu, Bo Shen, Tianye Niu, Xiaotong Hu, Xiujun Cai, Jihong Sun

**Affiliations:** ^1^ Department of Radiology, Sir Run Run Shaw Hospital, Zhejiang University School of Medicine, Hangzhou, China; ^2^ Department of Radiation Oncology, Rutgers-Cancer Institute of New Jersey, Rutgers-Robert Wood Johnson Medical School, New Brunswick, NJ, United States; ^3^ Department of Radiology, Hwa Mei Hospital, Key Laboratory of Diagnosis and Treatment of Digestive System Tumors of Zhejiang Province, University of Chinese Academy of Sciences, Ningbo, China; ^4^ Institute of Translational Medicine, Zhejiang University, Hangzhou, China; ^5^ Biomedical Research Center and Key Laboratory of Biotherapy of Zhejiang Province, Sir Run Run Shaw Hospital, Zhejiang University School of Medicine, Hangzhou, China; ^6^ Department of General Surgery, Innovation Center for Minimally Invasive Techniques and Devices, Sir Run Run Shaw Hospital, Zhejiang University School of Medicine, Hangzhou, China; ^7^ Innovation Center for Minimally Invasive Techniques and Devices, Zhejiang University, Hangzhou, China

**Keywords:** 5-year overall survival, advanced rectal cancer, prognostic model, radiomics, chemoradiation

## Abstract

Although rectal cancer comprises up to one-third of colorectal cancer cases and several prognosis nomograms have been established for colon cancer, statistical tools for predicting long-term survival in rectal cancer are lacking. In addition, previous prognostic studies did not include much imaging findings, qualitatively or quantitatively. Therefore, we include multiparametric MRI information from both radiologists’ readings and quantitative radiomics signatures to construct a prognostic model that allows 5-year overall survival (OS) prediction for advance-staged rectal cancer patients. The result suggested that the model combined with quantitative imaging findings might outperform that of conventional TNM staging or other clinical prognostic factors. It was noteworthy that the identified radiomics signature consisted of three from dynamic contrast-enhanced (DCE)-MRI, four from anatomical MRI, and one from functional diffusion-weighted imaging (DWI). This highlighted the importance of multiparametric MRI to address the issue of long-term survival estimation in rectal cancer. Additionally, the constructed radiomics signature demonstrated value to the conventional prognostic factors in predicting 5-year OS for stage II–III rectal cancer. The presented nomogram also provides a practical example of individualized prognosis estimation and may potentially impact treatment strategies.

## Introduction

The mortality rate of colorectal cancer has declined steadily over the last 2 decades, with Surveillance, Epidemiology, and End Results Program (SEER) data statistics showing 23.6% observed death rate in 1992 to 12.8% death rate in 2019 (https://seer.cancer.gov/statfacts/html/colorect.html). This is widely believed to be a consequence of improvements in surgical, medical, and supportive care (1). While substantial progress has been made, heterogeneity in survival outcomes exists (2–5). An accurate prognostication would be helpful to inform treatment decisions, determine clinical trial eligibility, and develop surveillance schedules. Tumor node metastasis (TNM) staging plays a vital role in predicting prognosis and facilitating treatment stratification, yet it is not sufficiently precise (6, 7). In the current TNM staging system, inclusion of tumor deposits (TDs) within nodal staging has given rise to worldwide discussions (8–11). While other important prognosis features, such as pretreatment serum level of carcinoembryonic antigen (CEA), extramural vascular invasion (EMVI), and circumferential resection margin (CRM), are acknowledged, they are not included in staging due to lack of standardized agreement or recommendations. Thus, a more precise survival estimation tailored to the individual patient is needed.

For colon cancer, there were established nomograms to predict recurrence or survival beyond the current TNM staging system with additional prognostic factors, both continuous and discrete, as well as nonlinear and complex mathematical relationships (12–14). In contrast, personalized prognostication of rectal cancer remains lacking despite more heterogeneous results. Valentini et al. (6) reported a nomogram incorporating clinical variables such as age, gender, TNM staging, chemoradiation, and surgical procedure to predict overall survival (OS). Similarly, van Gijn et al. (5) developed a nomogram to predict survival in patients treated with optional short-term radiotherapy by evaluating similar clinical variables and pathological TNM staging. Lately, Song et al. (15) extended the work with pretreatment/posttreatment CEA levels, Cancer Antigen (CA) 19-9 values, and combined clinical and pathological characteristics to predict OS of resected rectal cancer. However, these studies did not account for image findings, qualitatively or quantitatively.

Current improvements in survival estimation have largely been made due to advances in biologic and genomic technologies (16–20). However, the inability to obtain comprehensive information with regard to spatial and temporal heterogeneity continues to be a limitation in optimizing treatment strategy (21). Radiomics has been brought into the evolving topic, as it enables the noninvasive profiling of the disease (22, 23). Recent work in radiomics has provided insights in personalized medicine related to tumor detection, subtype classification, and therapeutic response assessment in rectal cancer (24, 25). Since imaging characteristics may also reveal underlying disease behavior and progression, in this study, we investigated whether a prognostic model that leverages the full complement of routinely available data elements and radiomics information could more accurately predict 5-year OS. We also established a radiomics nomogram to assess its incremental value to the traditional TNM system and clinical, histological, and radiological factors for individual long-term OS estimation in stage II–III rectal cancer.

## Methods

### Patients’ Characteristics

**Figure 1:**
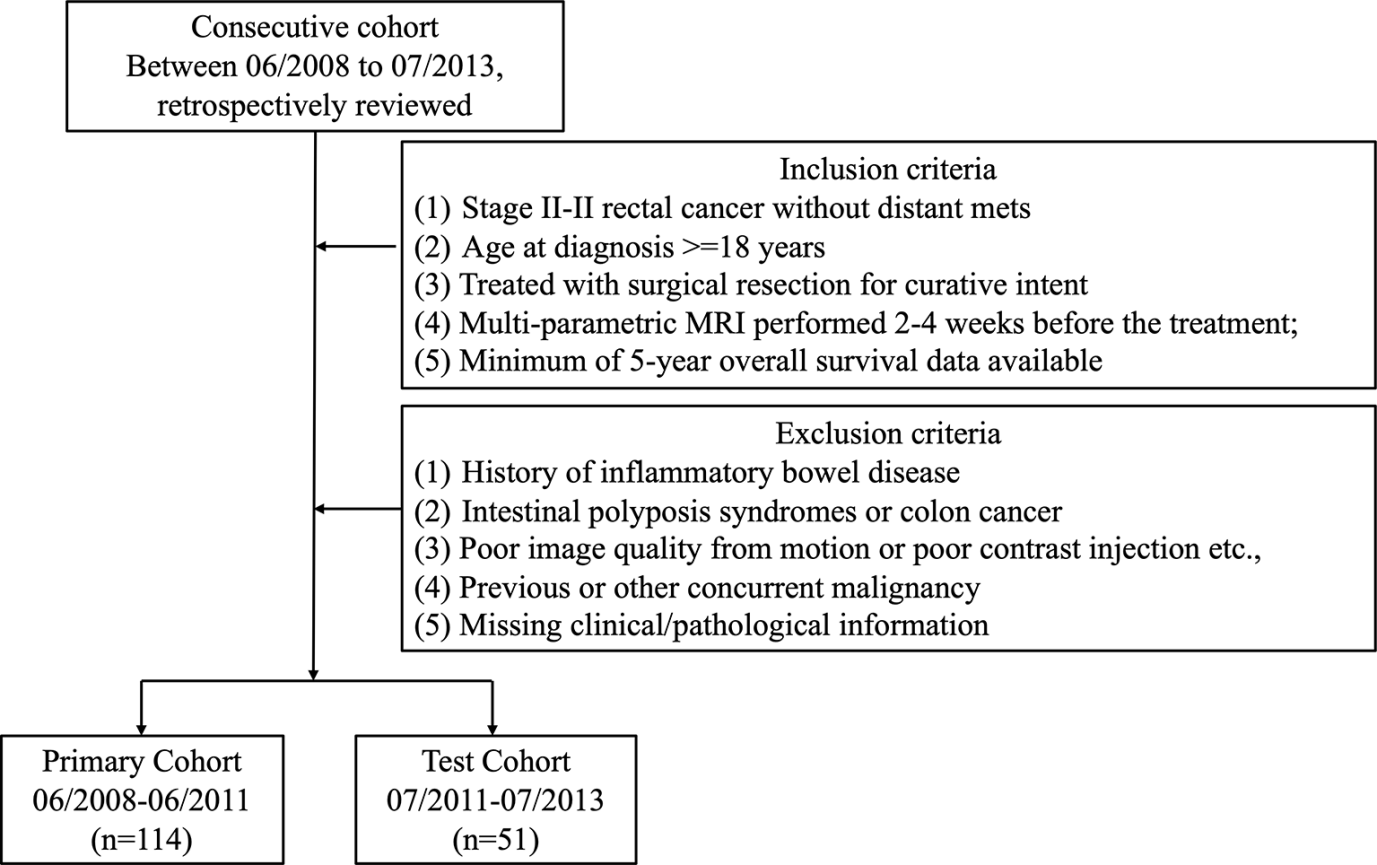
A flowchart showing patient selection.

Institutional Review Board (IRB) approval was obtained for this retrospective analysis, and informed consent was waived. All patients with rectal cancer [American Joint Committee on Cancer (AJCC) 7th edition] were reviewed from the institutional database starting from June 2008 to July 2013. All patients received Total Mesorectal Excision (TME) as standard treatment. Postoperative chemotherapy with either Folfox- or Xelox-based regimen was applied at the physician’s discretion. The criteria for inclusion in the study consisted of patients who 1) were diagnosed with stage II–III rectal cancer and no distant metastasis, 2) underwent surgical resection for curative intent, and 3) had a minimal of 5-year follow-up data. Patients with one or more of the following criteria were excluded from the study: history of inflammatory bowel disease, pelvic surgery, intestinal polyposis syndromes or colon cancer, low-quality MRI data from motion artifacts or poor contrast injection, concurrent malignancies, and missing clinical/pathological information. The patient selection flowchart is shown in **Figure 1**.

The recorded clinical and treatment data included age, gender, pretreatment CEA level, clinical tumor (cT) and node (cN) stage, postoperative TD status, surgical procedure [low anterior resection (LAR), abdominoperineal resection (APR), Hartmann’s procedure, or others], adjuvant chemotherapy (no/Folfox/Xelox). The radiological reading included tumor location from MRI (low, mid, and high) and tumor distance from the anal verge measured from colonoscopy. All images and reports were reviewed by two radiologists independently (with respective 10 and 8 years of experience in abdominal MRI), and the discrepancies were revisited until consensus has been reached.

Evaluation of the surgical resection specimen for residual tumor was performed under a standard reporting protocol in a central pathological laboratory at Sir Run Run Shaw Hospital. Two experienced gastroenterology pathologists independently reviewed the specimen, and a third expert pathologist was responsible for the final decision in case of a disagreement between the two pathologists. Data from pathological readings included tumor (yT) and nodal (yN) stages, total number of examined lymph node (LN), number of positive LN, positive lymph node ratio (LNR), and histology type (I-well, II-mid, or III-poorly differentiated, and IV-mucoid and signet ring cell carcinoma). The EMVI was scored as suggested by Smith et al. (26), with 0 if there was absence of invasion while 4 if with the most overt features of invasion. The final clinical endpoint was the long-term OS, which was defined as the time from the date of surgical resection until death. The minimum follow-up time to ascertain OS was 60 months. In total, 165 cases were identified (66 women; mean age 63 ± 12 years, range 19–89 years) with survival ranging from 5 to 121 months (median of 74 months).

### MRI Data Acquisition

Patients were scanned at the same institution with a 1.5 Tesla MR scanner (Signa Excite HD, GE Medical Systems) using 8-channel phased array body coil in supine position before curative treatment. Images included axial T1-weighted (T1w), high-resolution T2-weighted (T2w), four-phase dynamic contrast-enhanced MRI (DCE-MRI), and diffusion-weighted imaging (DWI) sequences.

**Figure 2:**
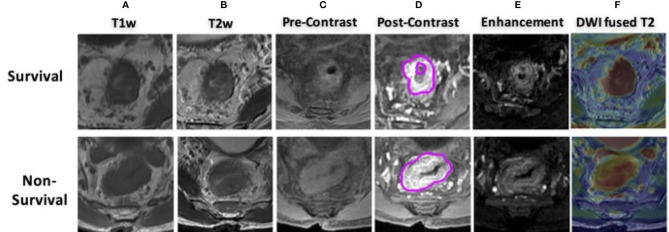
MR images of two male patients, both at 60 years old with mid-rectum cancer at stage cT3N+M0, pretreatment **(A)** T1-weighted image, **(B)** T2-weighted image, **(C)** precontrast image, **(D)** 60 s after contrast injection image, **(E)** subtraction image showing difference between panels **(D, C, F)** the derived apparent ADC map fused to a T2-weighted image. The first row shows a case as 5-year survival patient, and the bottom shows a non-survival case.

The acquisition parameters were as follows: axial T1w (T1w spin echo sequence, repetition time as TR/echo time as TE: 460/7.4 ms) and axial T2w (T2w fast spin echo sequence, TR/TE: 2,840/131 ms, image resolution 0.49 mm × 0.49 mm × 4 mm) maps were acquired. Then, multiphase T1w were obtained using a spoiled gradient echo sequence [liver acceleration volume acquisition (LAVA)]. Scan parameters were TR/TE 4.4/1.9 ms, flip angle 12°, bandwidth 325.5 kHz, image resolution: 0.7 mm × 0.7 mm × 2 mm. All patients were injected with 0.1 mmol/kg body weight Gd-DTPA at 2.5 ml/s. Contrast injection and data acquisition were trigged simultaneously. Four repetitions were acquired with one repetition before the injection of contrast agent (L1) and three at 15 s (L2), 60 s (L3), and 120 s (L4) after the injection. Axial DWI images were obtained by using single-shot echo planar imaging sequence (SSEPI; TR/TE 5,900/69.6 ms; image resolution: 0.98 mm × 0.98 mm × 5 mm; 2-mm intersection gap) with two b-factors of 0 and 800 s/mm^2^. The apparent diffusion coefficient (ADC) map was generated using these two DWI images with simple log-conversion. Patients’ examples with multisequence MR images are shown in **Figure 2**. Both were 60-year-old men with mid-rectum cancer at stage of cT3N+M0. One was 5-year survival, and the other was not. No significant differences were observed from qualitative visual inspection.

### Tumor Image Analysis

A region of interest (ROI) was delineated initially around the entire tumor by an experienced radiologist (10-year experience largely with colorectal MRI) using itk-SNAP software (www.itksnap.org) on each slice of T1w subtraction images (differences between the third-phase 60 s, after contrast injection and the first phase, before contrast injection), T2w, and DWI (b = 800 s/mm^2^). Then, images were transferred over to a Velocity workstation (Varian Medical Health, Palo Alto, CA) by aligning all sequences under the same frame. The delineated tumor was further adjusted using all other image sequences as references. To minimize the partial volume effect and effect of contouring variance, the segmented contour was eroded by 1 mm from the border and the remaining region was used for radiomics analysis. After 3 months, 30 patients in the training set were selected randomly and segmented again by him and another radiologist (with 8 years of experience) to assess intra/inter-reader agreement of the radiomics analysis using intraclass correlation coefficients (ICCs). We interpreted an ICC of 0.81–1 as almost perfect agreement, 0.61–0.80 as substantial agreement, 0.41–0.60 as moderate agreement, 0.21–0.40 as fair, and 0–0.2 as poor agreement. An ICC of greater than 0.6 was considered as satisfactory inter- and intra-reader reproducibility.

All images were preprocessed with z-score intensity normalization and resampled into isotropic resolution (1 mm^3^ × 1 mm^3^ × 1 mm^3^) with gray level quantized to 64 gray level. The radiomics analysis was performed using an in-house built program. Eight sequences as T1w, T2w, ADC map, L2, L3, L4, L2–L1, L3–L1, L4–L1) were analyzed. A total of 4,686 radiomics features from the category of morphology-, histogram-, texture-, and wavelet-based features were extracted from each individual patient: 1) For morphology features, 6 shape descriptors as volume, surface area, circularity, compactness, convexity, and irregularity were included; 2) 14 histogram-based features included min, mean, max, 90, 80, …, 10 percentiles, skewness, kurtosis; 3) 51 texture-based features included 7 gray-level run length matrix (GLRLM), as short run emphasis (SRE), long run emphasis (LRE), gray-level non-uniformity (GLN), run percentage (RP), run length non-uniformity (RLN), low gray-level run emphasis (LGRE), high gray-level run emphasis (HGRE). In this study, 44 gray-level co-occurrence matrix (GLCM)-based texture features were also collected with two distances as 1 and 2 pixels with each along 13 directions in 3D; 4) In addition, a discrete wavelet transform was used to decompose volumetric images into eight decomposed images, labeled as LLL, LLH, LHL, LHH, HLL, HLH, HHL, and HHH, where L and H are low- and high-frequency filters, respectively. For example, HHL represents a decomposition volume with high-pass filtering on X- and Y-directions and low-pass filtering along the z-direction. In each decomposed volume, histogram-based and texture-based features were extracted, resulting in a total of 520 (8 × 65) wavelet-transformed features for each sequence. In the end, a total of 4,686 (6 morphology+14 × 8 + 51 × 8 + 520 × 8) radiomics features were obtained from each patient.

### Statistical Analysis

The patients treated from June 2008 to June 2011 (n = 114) were selected as a primary cohort, which was further randomly assigned into training and validation data sets with 4-fold cross-validation. The patients treated from July 2011 to July 2013 (n = 51) were assigned as a separate test cohort. The differences between OS vs. non-OS groups in both primary and test cohorts were compared using an analysis of variance (ANOVA) for continuous variables and Fisher’s exact test for categorical variables. Multivariate analysis with a Cox regression analysis model was performed to detect independent prognostic factors for long-term survival.

Feature selection was performed in 3 steps to select the optimal survival-related features using the training cohort. Firstly, linear correlation between each pair features was evaluated using the Spearman test. Redundant features with linear correlation coefficient (arbitrary set) >0.90 were removed. Then, the least absolute shrinkage and selection operator (LASSO) regression algorithm was used for feature selection and model construction. LASSO regression shrinks the coefficient of unrelated feature toward zero, and related parameters are retained. The robustness of feature selection was tested by conducting a 4-fold cross-validation 100 times with binomial deviance minimization criteria from the primary cohort. Regression coefficients were estimated by LASSO. Lastly, the selected imaging features were then combined into a radiomics score through a linear combination of selected features weighted by their respective LASSO regression coefficients.

To further demonstrate the incremental values of radiomics signature to traditional risk factors, the discrimination performance between survival and non-survival groups was assessed with 1) TNM staging, 2) clinical-radiological (non-radiomics) features, 3) radiomics signature, and 4) combined all information. Discrimination was demonstrated by a receiver operating characteristic (ROC) curve in both primary and testing cohorts (with 1 indicating perfect prediction and 0.5 as no better concordance than chance). To provide clinicians a quantitative tool in predicting individual probability of 5-year OS, a nomogram was built based on multivariate logistic analysis in the primary cohort. Harrell’s C-index was used to measure the nomogram discriminatory performance. Calibration curves accompanied by the Hosmer–Lemeshow test were used to assess the model fitting. A diagonal line along the calibration curve represented perfect agreement, and a significant p-value suggested a non-good fitting.

## Results

### Clinical Characteristics

The present study included 165 patients with a mean age of 67 ± 13 years [standard deviation (SD)], range 19–89 years. All patients had minimal 60-month and up to 121-month follow-up. Patient characteristics are given in **Table 1**. As of the last follow-up, 123 patients (75%) experienced a confirmed 5-year (equal to or over 60-month) OS. Our data showed that OS for stage IIA was 80%, 67% for IIB, 78% for IIIA, 75% for IIIB, and 55% for IIIC.

**Table 1:** Patient Characteristics.

	Primary			Testing		
	5-yr OS (88)	5-yr Non-OS (26)	P-value	5-yr OS (35)	5-yr Non-OS (16)	P-value
*Survival Months*	82±15 [60,121]	30±15 [5,59]		82±13 [60,104]	36±14 [13,59]	
***Clinical Data***						
Age (years)	64±12 [29,84]	66±10 [45,80]	P=0.34	59±12 [19,89]	69±13 [40,89]	P=0.08
Gender						
* Male*	57	9	P=0.01*	23	10	P=0.8
* Female*	31	17		12	6	
Pre-tx CEA	7.3±15 [0.6,102]	26±46 [1.3,166]	P=0.04*	8.0±9.5 [1.1,43.5]	22±37 [2,116]	P=0.04*
cT stage						
* T2*	16	5	P=0.81	8	3	P=0.92
* T3*	57	17		26	13	
* T4*	15	4		1	0	
cN stage						
* N0*	54	15	P=0.32	22	8	P=0.06
* N1*	32	8		11	8	
* N2*	2	3		2	0	
Stage						
* IIA*	34	7	P=0.02*	18	6	P=0.07
* IIB*	1	1		1	0	
* IIIA*	3	1		4	1	
* IIIB*	40	9		11	8	
* IIIC*	10	8		1	1	
Tumor Deposit	22/50	14/23	P=0.05*	8/25	9/14	P=0.04*
***Treatment Data***						
Surgery						
* LAR*	14	10	P=0.01*	25	7	P=0.05*
* APR*	14	7		6	4	
* Hartmann’s*	10	6		3	4	
* Others*	5	3		1	1	
Post-operative Chemo						
None	40	16	P=0.2	7	8	P=0.06
Folfox	32	6		18	5	
Xelox	16	4		10	3	
***Radiological Data***						
Location						
* Lower*	12	7	P=0.23	6	2	P=0.86
* Mid*	42	12		20	11	
* High*	34	7		9	3	
Dist from Anal Verge	7.8±2.9 [4,16]	8.9±4.1 [1.6,17]	P=0.21	8.8±4.2 [3,15]	10.5±4.5 [4,20]	P=0.21
EMVI						
* 0*	38	6	P=0.8	1	1	P=0.9
* 1*	2	0		2	1	
* 2*	2	1		1	1	
* 3*	36	11		1	0	
* 4*	10	8		30	13	
						
***Histo-Pathological Data***						
yT stage						
* T2*	4	3	P=0.3	5	1	P=0.08
* T3*	79	21		29	12	
* T4*	5	2		1	3	
yN stage						
* N0*	40	6	P=0.04*	19	5	P=0.04*
* N1*	37	14		13	7	
* N2*	11	6		3	4	
Total LN	16±7 [3,37]	13±7 [5,28]	P=0.06	16±6 [7,33]	13±5 [8,29]	P=0.21
Positive Nodes	2±3 [0,10]	3±4 [0,13]	P=0.13	1.3±2.5 [0,8]	1.7±2.0 [0,11]	P=0.11
Positive LN Ratio	0.1±0.2 [0,0.8]	0.2±0.2 [0,0.7]	P=0.04*	0.08±0.17 [0,0.5]	0.16±0.13 [0,0.7]	P=0.04*
Histology Type						
* I-well differentiated*	34	9	P=0.9	14	4	P=0.6
* II-mid differentiated*	44	14		16	10	
* III-poor differentiated*	4	2		1	0	
* IV-mucoid or signet ring*	6	1		4	2	
***Radiomics Data***						
Radiomics score	0.16±0.18 [-0.32,0.82]	-0.06±0.13 [-0.36,0.21]	P<0.001*	0.16±0.19 [-0.04,0.72]	-0.05±0.11 [-0.23,0.14]	P<0.001*

OS, overall survival; Pre-tx CEA, pre-treatment carcinoembryonic level; cT-stage, clinical tumor stage; cN-stage, clinical node stage; APR, abdominoperineal resection; LAR, lower anterior resection; Dist, Distance; yT, pathological T-stage; yN, pathological N-stage; Total LN, total lymph node; Positive LN Ratio, positive lymph node ratio; EMVI, extramural vascular invasion; *: p≤0.05;

All patients had formal rectal resections, 101 (61%) underwent LAR, 31 (19%) received APR, 23 (14%) had a Hartmann’s procedure, and 10 (6%) received other types of resections. Patients who underwent LAR had the best prognosis, while those who underwent a Hartmann’s procedure had the worst prognosis (p ≤ 0.05). The presence of TDs (at the time of postsurgical evaluation) was also found to be associated with a poorer outcome as decreased OS.

A total of 95 patients (58%) had pathologically confirmed nodal involvement (43% with yN1 and 15% with yN2). Both nodal involvement and increasing node stage were significantly associated with poorer OS in both training and validation data sets (p < 0.05). The 5-year OS survival rate with yN0 is 84%, yN1 is 70%, and yN2 is 58% among all patient cohorts. The median number of examined lymph nodes per patient was 15 (range 3–37). Metastatic nodes were presented in 89 patients (53.9%). A positive LNR was found strongly correlated with poorer OS (p < 0.04).

### Construction and Validation of Radiomics Signature

**Figure 3:**
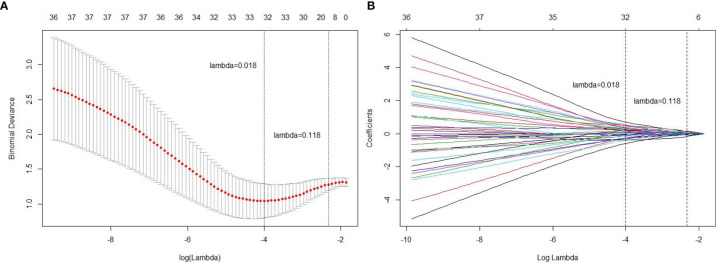
Radiomics feature selection using the least absolute shrinkage and selection operator (LASSO) regression model. **(A)** Tuning parameter (lambda) selection in the LASSO model used 4-fold cross-validation *via* minimum criteria. **(B)** A coefficient profile plot was produced against the log (lambda) sequence. Vertical line was drawn at the value selected using 4-fold cross-validation, where optimal lambda resulted in 8 non-zero coefficients (features).

To avoid overfitting, the selected feature was limited to within 10:1 ratio relative to 114 patients in the primary cohorts, which means less than 11 features should be selected (27). **Figure 3** shows tuning parameter (λ) for feature selection with the values of coefficients closer to 0 with higher lambda. To keep the minimum binominal deviance, the number of features should be 32. Yet considering the number constraint of training cases, we decided to select 8 features that provided satisfactory performance and did not increase much denominal deviance. The radiomics features with a non-zero coefficient in the LASSO Cox regression model were as follows: LAVA2(Hist_Skewness), LAVA3(HLL_HistMax), LAVA3(GLCM_DiffrenceEntropy), T1w(LongRunEmphasis), T1w(HLL_HistMax), T2w(LLL_Hist10%), T2w(HLH_Hist40%), and ADC (GLCM_InfMeasCorr). Among the 8 selected features, 3 were from DCE-MRI LAVA sequences, 4 were from anatomical T1w or T2w images, and 1 was from DWI ADC map. The radiomics signature was constructed with relative weightings directly generated from LASSO regression (28). A Rad_score was calculated using the following formula:


$$\begin{array}{c} \text{Rad}\_\text{score}=\text{sigmoid}[-0.664+0.106*\text{LAVA}2\left({\text{Hist}}_{\text{Skewness}}\right)-0.029\\ \;*\text{LAVA}3\left({\text{HLL}}_{\text{HistMAx}}\right)+0.022*\text{LAVA}3\left({\text{GLCM}}_{\text{DifferenceEntropy}}\right)+0.067\\ \;*\text{T}1\text{w}\left(\text{LongRunEmphasis}\right)+0.05*\text{T}1\text{w}\left({\text{HLH}}_{\text{HistMax}}\right)-0.082\\ \;*\text{T}2\text{w}\left(\text{LongRunEmphasis}\right)+0.05*T2\left({\text{HLH}}_{Hist40}\%\right)-0.092\\ \text{ ADC}\left({\text{GLCM}}_{\text{InfMeasCorr}}\right)] \end{array}$$

where sigmoid(x) = [1+exp(-x)]=^-1^

**Figure 4:**
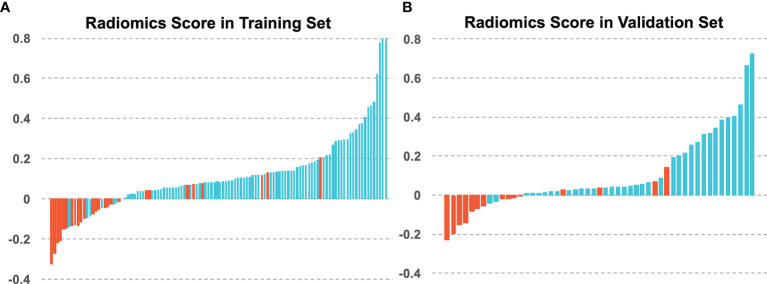
The radiomics scores of each patient in **(A)** the training set and **(B)** the validation set, with blue for 5-year OS and red for 5-year non-OS patients.

Higher Rad-score patients generally had longer survival compared to those with lower scores. Distributions of the Rad_score in both primary and test cohorts are given in **Figure 4**. There was a significant difference in the Rad-score between 5-year OS vs. non-OS group in the primary cohort (p < 0.001*), which was also confirmed in the test cohort (p < 0.001*).

### Incremental Prognostic Value of Radiomics to TNM Staging and Clinical-Histological-Radiological Features

**Figure 5:**
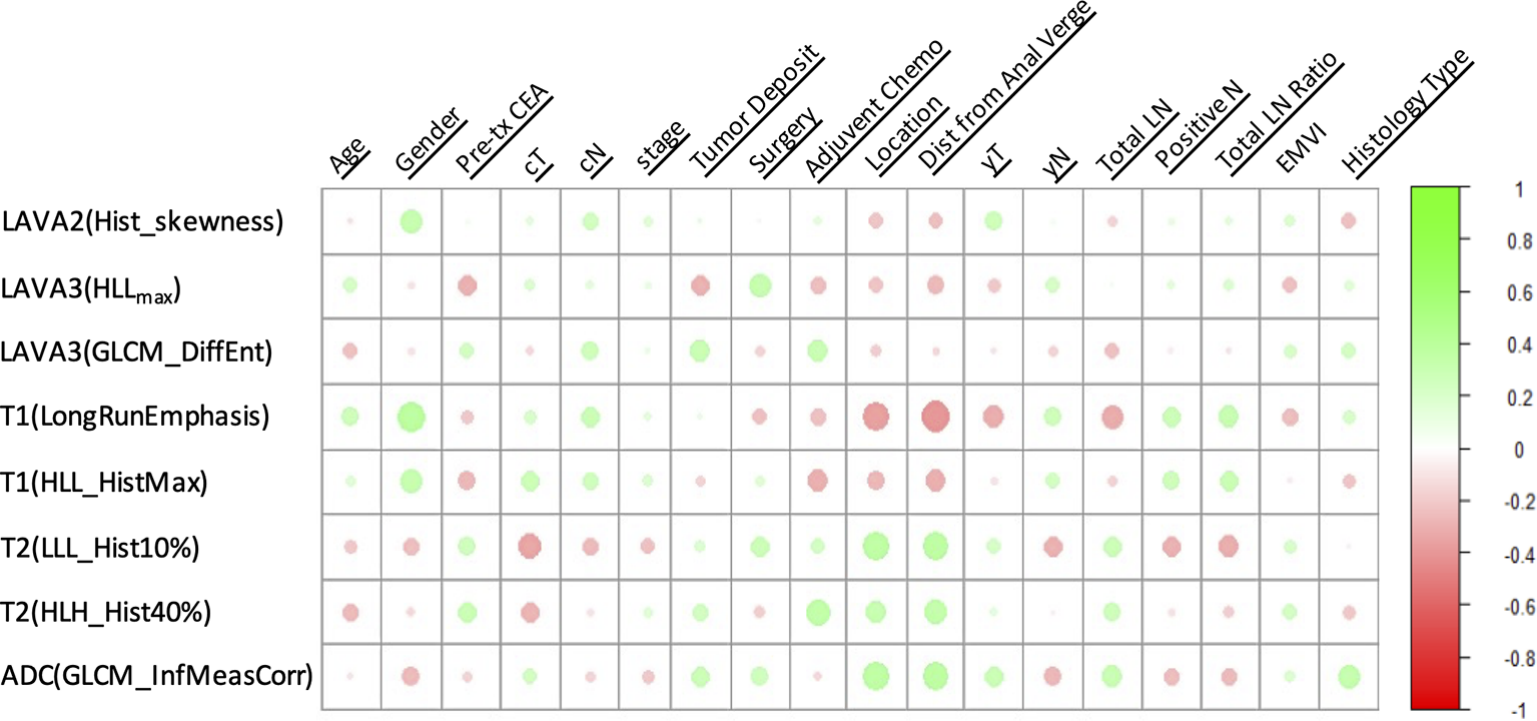
A heatmap showing the correlation between selected radiomics features and clinical-histological-radiological parameters, with green as positive correlation and red as negative correlation.

**Figure 6:**
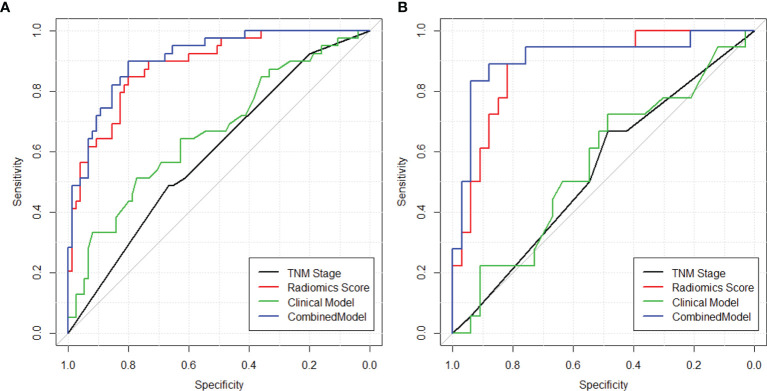
ROC curves showing the prognostic power in estimating 5-year OS in both **(A)** training and **(B)** validation sets using (1) TNM staging (2), clinical model with clinical-histopathological-radiological (non-radiomics) risk factors, (3) radiomics score, and (4) combined model with both radiomics and non-radiomics information.

The heatmap showing the correlation between the selected radiomics features and the clinical-histological-radiological factors is shown in **Figure 5**. For example, higher ADC(GLCM_InfMeasCorr) was associated with higher histology type. Most of the selected radiomics features showed a correlation with pretreatment CEA levels and adjuvant chemotherapy status. To further demonstrate the incremental prognostic value of radiomics to conventional prognostic features, four risk models in total were built: 1) TNM staging, 2) clinical-histological-radiological (non-radiomics), 3) radiomics signature, and 4) combined all selected features. The prognostic power in estimating 5-year OS in both primary and test data set was illustrated with ROC curves in **Figure 6**. The AJCC TNM staging system had the lowest area under the ROC curve (AUC) of 0.60 [95% confidence interval (CI): 0.50, 0.70] in the primary cohort and 0.54 (95% CI: 0.38, 0.69) in the test cohort, and the non-radiomics clinical model had a higher AUC of 0.66 (95% CI: 0.56, 0.77) and 0.56 (95% CI: 0.39, 0.73) in the primary set and test set, respectively. The radiomics signature yielded an AUC of 0.88 (95% CI: 0.82, 0.95), and a similar trend was observed in the test cohort with an AUC of 0.89 (95% CI: 0.79, 0.98). The combined model showed the best prognosis of survival outcome with an AUC of 0.91 (95% CI: 0.85, 0.96) in the primary set and 0.91 (95% CI: 0.81, 0.99) in the test set.

**Figure 7:**
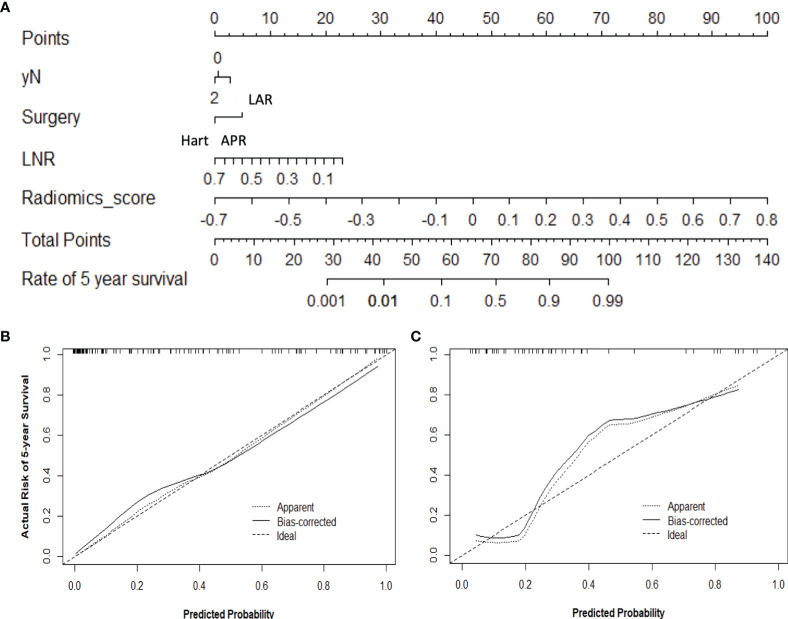
Use of the constructed nomogram to estimate 5-year OS probability, along with the assessment of the model calibration, **(A)** nomogram with combined radiomics and non-radiomics clinical information, and calibration curves for the nomogram in the **(B)** training and **(C)** validation data sets. The y-axis represents the actual rate, and the x-axis represents the predicted probability of 5-year OS, with the diagonal line representing a perfect prediction. The solid line represents the performance of the radiomics model, of which a closer fit means better prediction.

**Figure 8:**
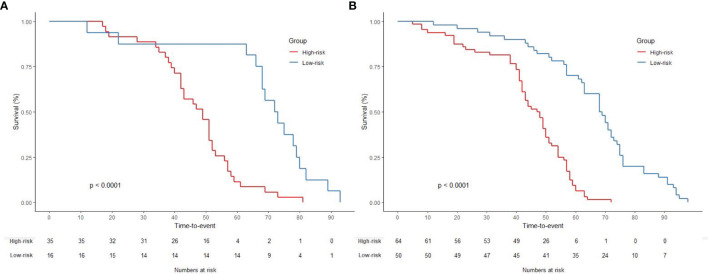
Graphs show results of Kaplan–Meier survival analyses according to the radiomics signature for patients in **(A)** the primary data set (left) and those in **(B)** the test data set (right). A significant association of the radiomics signature with the OS was shown in the primary data set, which was then confirmed in the test data set.

Furthermore, a nomogram with combined clinical-histopathological-radiological and radiomics features was developed in assessing the 5-year OS probability. The resulting nomograms can estimate outcome probability by assigning a score (upper scale) to each predictor value. The probability for 5-year OS (bottom scale) was a sum of these scores. The radiomics signature was the most important factor, followed by a positive LNR, surgery type (LAR vs. APR vs. Hartmann’s vs. others), and pathological node status (yN). The final nomogram with calibration curves is illustrated in **Figure 7**. The C-index for the model was 0.898 (95% CI: 0.832, 0.947) within the primary cohort and 0.901 (95% CI: 0.803, 0.987) for the test cohort. The Hosmer–Lemeshow test yielded a nonsignificant statistic (p = 0.83), suggesting no departure from the perfect fit in the primary cohort. Good performance was also observed for the probability of pCR in the testing cohort with a nonsignificant statistic (p = 0.54). Patients were further classified into a high-risk group and a low-risk group with cutoff point identified on the ROC curve. Kaplan–Meier estimate of event rates over time showed statistically different outcomes for OS (p < 0.001). Results are also confirmed in the test data set as shown in **Figure 8**.

## Discussion

In this study, we extended the 5-year OS prediction beyond TNM schema to include clinical, histopathological, radiological, and high-throughput radiomics information. The combined nomogram outperformed the TNM staging or a clinical system, which demonstrated the incremental value of the radiomics signature in individualized OS association in patients with stage II–III rectal cancer. Incorporating the radiomics signature and clinically available prognostic factors into an easy-to-use nomogram also allowed the model to support decision-making in daily practice, while further external validation is needed.

Despite rectal cancer comprising up to one-third of colorectal cancer cases and several prognosis nomograms having been established for colon cancer (13, 19), statistical tools for predicting long-term survival in rectal cancer are limited. Although MRI has become an indispensable tool for diagnosis by guiding treatment decisions for rectal cancer, the studies did not account for MRI findings. As such, in this study, we incorporated radiologists’ qualitative assessment such as the depth of tumor spread beyond the muscularis propria, EMVI, and TDs into survival prediction. In addition, we extracted quantitative features from full-panel multiparametric MRIs with joint T1w, T2w, DCE-MRI, and DWI information and constructed a radiomics score for prognosis estimation. It is noteworthy that the identified radiomics signature consisted of three from dynamic MRI, four from anatomical MRI, and one from functional DWI. This study underlines the importance of multiparametric MRI in quantitative format to address the issue of long-term survival estimation in rectal cancer.

Pooling radiomics features to predict treatment outcome is still a relatively new concept. A few related studies that have been done focused on short-term treatment response as pathological complete response (pCR) to neoadjuvant chemoradiation therapy (CRT) (24, 25, 30). Although preoperative CRT has been demonstrated to improve local control, a recent meta-analysis and several clinical trials showed that there were no survival benefits for patients with stage II–III disease (2, 4, 7, 31, 32). This suggests that previously derived radiomics models may have limitations in estimating long-term clinical outcome. Instead, our study extends the “-omics”-based analysis to OS estimation, with all patients having a minimum of 60-month follow-up. In addition, unlike prior prognostic investigations that mostly analyzed patients with all stages of disease, our study focused exclusively on patients with stage II–III cancer. It is notable that when patients were stratified by clinical stage, there was a difference in OS for stage IIA vs. IIB, and stage IIIA vs. IIIB or IIIC, which suggested that heterogeneity existed in the survival outcomes. Our results showed that this radiomics signature was able to stratify patients into different risk levels beyond TNM staging or conventional clinical prognostic models. This is consistent with our current knowledge of cancer, in which malignant tumors consist of heterogeneous cell populations with distinct molecular and microenvironmental differences, increasing the likelihood of developing resistance to treatment and resulting in metastases (33, 34). In contrast, the traditional TNM staging or clinical assessment is based on gross anatomy information, with minimal regard for intratumor heterogeneity. With medical imaging, radiomics can extract features from the imaging characteristics of the entire tumor that provides a robust way to characterize the intratumor heterogeneity noninvasively.

Developing nomograms have been considered helpful in oncology prognosis (32, 35). In our study, a nomogram was built by combining selected features into a final score representing the probability of 5-year OS. Among them, the radiomics signature has the highest contribution, followed by positive LNR, types of surgery, and pathological N-staging. Increasing N-stage, as confirmed by previous studies, was found to be associated with poor survival (6, 36). The number of positive node/number of total nodes, which was revealed to be the most important risk factor of 5-year OS for colon cancer based on the well-known model from Weiser et al. (14), was only recently proposed for rectal cancer, but evidence is still limited (8). Our study confirmed that a positive LNR was an independent prognostic factor. Regarding the surgical type, our study patients with LAR operations had better survival outcomes compared to those receiving APR, as illustrated in the nomogram. There were conflicting reports regarding the prognostic effect of surgical types on OS rate. While most reported improved OS rates have been observed for LAR compared to APR (14, 36, 37), a few studies found no significant differences (38). Some previous studies also showed that the 5-year OS was higher for patients who had an LAR compared to those who had a Hartmann’s procedure (36). The decision to perform a Hartmann’s procedure was likely related to the individual patient characteristics such as severe cardiorespiratory or renal disease, which might impact survival. Interestingly, age was not found significantly correlated to OS. The reason for this is not immediately clear due to limited patient numbers. Age differences existed between good- and poor-survival group in the test cohort but not in the primary cohort. Since the model was developed using the primary cohort, age was not chosen as a predictor in this study. Other conventionally confirmed prognostic risk factors, such as pretreatment CEA level, TNM staging, TDs (39), were all found to be statistically different between better survival and worse survival groups, yet they were not selected into the final model as they were less important compared to radiomics signature.

To develop the radiomics signature, a total of 4,686 features were reduced to a set of only 8 potential descriptors using a LASSO regression model. In this method, the regression coefficients of most features were shrunk toward zero during model fitting, allowing identification of features that were strongly associated with OS. More importantly, it allowed radiomics signatures to be constructed into a generalized linear model. Recently, advanced machine learning-based methods have been used, yet the complexity of U-net-based deep learning or conventional neural network (CNN) typically requires intensive computation of the input, thus requiring a large sample set. Not surprisingly, Shi et al. (40) reported that the prediction power of deep learning methods does not necessarily outperform conventional logistic regression in pCR prediction on a data set with 51 locally advanced rectal cancer patients. Other feature selection methods such as support vector machine or other deep learning-based algorithms, although beyond the scope of this paper, may be explored to further improve model performance (41). Moreover, by constructing multilayer nonlinear complex relationships, deep learning methods are more like a “black-box,” making it difficult for physicians to interpret the association of the input images to outcome. In contrast, not only does LASSO surpass the method of choosing predictors based on the strength of their association with outcome, but it also enables the panel of selected features to be combined into a radiomics score. Nevertheless, the linear combination of selected features weighted by their respective coefficients provides an intuitive tool for the clinicians to interpret the results.

Limitations of this study included the lack of external validation with the retrospective nature of data collection. A large-scale prospective multicenter validation cohort is warranted to assess the generalizability of the reported findings; however, the protracted length of a longitudinal cohort with long-term survival data may make the research daunting. On the other hand, the statistical analysis with cross-validation used in this study justified that the identified radiomics model could hold great potential for clinical application in postoperative outcome estimation. Another limitation is that the radiological assessment by two radiologists was performed in consensus; it was impossible to assess the inter-reader agreement. Additionally, the tumor ROIs were manually performed, which was time-consuming and subjected to operators’ variations. Semi- or full automatic segmentation may be needed in the future to improve the robustness of radiomics feature extraction. Moreover, due to the retrospective nature of this study, some important prognostic features such as margin positivity were not collected nor analyzed. Lastly, we only used LASSO for feature selection and model construction. It is known that L1 norm regularization can get sparse results. The variance of the model can be reduced using a higher lambda value, but it also results in a smaller number of features selected, thus leading to a biased model. Bias may be improved if using L2 norm instead. However, it will result in a large number of features selected, which leads to an overfitting. Other feature selection algorithms such as bridge and decision tree warrant to be further explored to achieve the best robust model.

Overall, the fact that clinicians naturally integrate multiple manifestations of disease to make an estimation and determine consequent therapy rather than focus on a single symptom underscores the necessity of multivariable estimation. Our study supported the suggestion that multiple variables could provide a more statistically meaningful approach to address the issue of long-term survival estimation using a multicomponent radiomics signature. The combined model with clinical-radiological-histological and radiomics signature yielded the highest AUC with 0.91 (95% CI: 0.85, 0.96) compared to any other models with TNM staging or clinical prognostic factors. The proposed nomograms were also well calibrated with nonsignificant p-values from Hosmer–Lemeshow test. Future work will include a large multi-institute prospective trial to validate the generalizability of the proposed model. Deep learning-based automatic segmentation is also under development to facilitate robust and efficient radiomics feature extraction. Nevertheless, although the usefulness of the proposed nomogram lacked external validation, the combined model, which considers multiple risk factors, was imperative. These findings and the nomogram may help patients with potential shorter OS to receive more aggressive treatment plans. Thus, our study may present a more efficient and integrated tool that enables earlier personalized treatment.

## Data Availability Statement

The software code used to analyze the current study is available upon request.

## Ethics Statement

The studies involving human participants were reviewed and approved by the Department of Radiology, Sir Run Run Shaw Hospital, Zhejiang University School of Medicine. The patients/participants provided their written informed consent to participate in this study.

## Author Contributions

Conception and design: KN, YZ, XH, XC, and JS. Development of methodology: KN, YZ, PH, and PY. Analysis and interpretation of data: KN, YZ, PY, and JS. Writing, review, and/or revision of the article: KN, PH, JZ, PY, SJ, NY, XD, SX, BS, TN, XH, XC, and JS. All authors contributed to the article and approved the submitted version.

## Funding

This study was supported by the National Key Research and Development Program of China (2016YFA0100900), the National Natural Science Foundation of China (81871403), the Key Research and Development Program of Zhejiang Province (2019C03014), and the Fundamental Research Funds for the Central Universities and research grant support from Varian Inc.

## Conflict of Interest

The authors declare that the research was conducted in the absence of any commercial or financial relationships that could be construed as a potential conflict of interest.

## Publisher’s Note

All claims expressed in this article are solely those of the authors and do not necessarily represent those of their affiliated organizations, or those of the publisher, the editors and the reviewers. Any product that may be evaluated in this article, or claim that may be made by its manufacturer, is not guaranteed or endorsed by the publisher.
